# Abnormal baseline brain activity in drug-naïve patients with Tourette syndrome: a resting-state fMRI study

**DOI:** 10.3389/fnhum.2013.00913

**Published:** 2014-01-02

**Authors:** Yonghua Cui, Zhen Jin, Xu Chen, Yong He, Xia Liang, Yi Zheng

**Affiliations:** ^1^Department of Pediatrics, Beijing An Ding Hospital, Capital Medical UniversityBeijing, China; ^2^Department of Medical Imaging, 306 Hospital of People’s Liberation ArmyBeijing, China; ^3^State Key Laboratory of Cognitive Neuroscience and Learning and International Digital Group/McGovern Institute for Brain Research, Beijing Normal UniversityBeijing, China; ^4^Center for Collaboration and Innovation in Brain and Learning Sciences, Beijing Normal UniversityBeijing, China; ^5^Neuroimaging Research Branch, National Institute on Drug Abuse, National Institutes of HealthBaltimore, MD, USA

**Keywords:** Tourette syndrome, resting-state fMRI, amplitude of low-frequency fluctuation, fractional amplitude of low-frequency fluctuation, cortico-striatal-thalamo-cortical

## Abstract

Tourette syndrome (TS) is a childhood-onset chronic disorder characterized by the presence of multiple motor and vocal tics. This study investigated spontaneous low-frequency fluctuations in TS patients during resting-state functional magnetic resonance imaging (rs-fMRI) scans. We obtained rs-fMRI scans from 17 drug-naïve TS children and 15 demographically matched healthy children. We computed the amplitude of low-frequency fluctuation (ALFF) and fractional ALFF (fALFF) of rs-fMRI data to measure spontaneous brain activity, and assessed the between-group differences in ALFF/fALFF and the relationship between ALFF/fALFF and tic severity scores. Our results showed that the children with TS exhibited significantly decreased ALFF in the posterior cingulate gyrus/precuneus and bilateral parietal gyrus. fALFF was decreased in TS children in the anterior cingulated cortex, bilateral middle and superior frontal cortices and superior parietal lobule, and increased in the left putamen and bilateral thalamus. Moreover, we found significantly positive correlations between fALFF and tic severity scores in the right thalamus. Our study provides empirical evidence for abnormal spontaneous neuronal activity in TS patients, which may implicate the underlying neurophysiological mechanism in TS and demonstrate the possibility of applying ALFF/fALFF for clinical TS studies.

## INTRODUCTION

Tourette syndrome (TS) is a childhood-onset chronic disorder characterized by the presence of involuntary, multiple movements and vocalizations called “tics” which last for more than one year. TS is a common disorder with prevalence in between 0.05 and 3% (Freeman et al., 2000) and could cause impairments such as distress, interference with activities, social impact, occupational difficulty, and occasional physical morbidity (Leckman et al., 2006).

Mink (2001) proposed a model of dysfunction of the cortico-striatal-thalamo-cortical (CSTC) circuit in TS, hypothesizing that aberrant activity in the striatum will release the thalamus from inhibition, and the thalamus will excite the cortex to the execution of unwanted movements (tics). Evidence for abnormalities in subcortical and cortical regions within the CSTC circuits has been derived from structural and functional neuroimaging studies in TS (for a review, see Martino and Leckman, 2013). Structural imaging studies have revealed consistent findings of reduced volume in children and adults with TS in striatal areas, particularly the caudate nucleus (Peterson et al., 1993, 2003; Hyde et al., 1995; Makki et al., 2008), with a smaller caudate volume in childhood would lead to higher symptom severity later in life (Bloch et al., 2005). Cortical thinning in sensorimotor cortices has been found in children and adults with TS, with a negative relationship to tic severity (Sowell et al., 2008; Fahim et al., 2010; Worbe et al., 2010). Functional imaging studies have revealed increased activity in the supplementary motor area (SMA) prior to tics (Bohlhalter et al., 2006; Hampson et al., 2009), which has been suggested as reflecting the premonitory urge that precedes tics, and might give rise to the aberrant striatal activity, which has been demonstrated in positron emission tomography (PET) and single photon emission tomography (SPET) studies to be increasing along with blood flow in TS subjects during tic release (Lerner et al., 2007). Moreover, a previous study has found aberrant activity in regions within the cognitive control network (Church et al., 2009), expanding the functional anatomy of putative abnormalities in TS from the CSTC circuit to other cortical networks that support cognitive functions.

However, most results from functional magnetic resonance imaging (fMRI) studies are generally inconsistent, which could be due to the heterogeneities in sample and task across studies (Martino and Leckman, 2013). Resting-state fMRI (rs-fMRI), first proposed by Biswal et al. (1995), measures the physiologically meaningful spontaneous low-frequency (typically 0.01–0.1 Hz) fluctuations (LFFs), providing opportunities to investigate the baseline brain activity free of task differences. With the advantages of easy application (compared to conventional task-driven paradigms), rs-fMRI has been widely applied to study brain function under normal and pathological conditions (Fox and Raichle, 2007; Zhang and Raichle, 2010). However, as far as we know, there are very few fMRI studies investigating the TS-related alterations in baseline brain activity that could help us to understand the underlying pathophysiological characteristics of TS (Church et al., 2009).

Amplitude of LFF (ALFF) and fractional ALFF (fALFF) measure the ALFF of the fMRI signal and have been used to characterize the regional spontaneous neuronal activity (Raichle and Mintun, 2006; Zang et al., 2007). ALFF is defined as total power within the low-frequency range, typically 0.01–0.1 Hz (Zang et al., 2007), whereas fALFF measures the power within the low-frequency range divided by the total power in the entire detectable frequency range (Zou et al., 2008). ALFF has higher test–retest reliability in gray matter regions than fALFF, while fALFF is more specific than ALFF and is more effective at minimizing artifactual contributions of cardiac and respiratory (Zuo et al., 2010). Both ALFF and fALFF have been successfully applied to detect the abnormal spontaneous brain activity of various neuropsychiatric disorders, such as mild cognitive impairment (MCI; Han et al., 2012), bipolar depression (Liu et al., 2012), Alzheimer’s disease (AD; Wang et al., 2011b), schizophrenia (Hoptman et al., 2010), and mesial temporal lobe epilepsy (Zhang et al., 2008). Aberrant ALFF/fALFF have also been found in patients with attention deficit hyperactivity disorder (ADHD; Zang et al., 2007; Yang et al., 2011) and obsessive–compulsive disorder (OCD; Hou et al., 2012), which are two common comorbid disorders of TS.

In this study, we computed ALFF and fALFF from rs-fMRI data to investigate the alterations in spontaneous brain activities in children with TS compared with demographically matched healthy children. We sought to determine (i) whether the children with TS show abnormal ALFF/fALFF, and (ii) whether the ALFF/fALFF abnormalities correlate with their tic severity scores.

## MATERIALS AND METHODS

### SUBJECTS

Tourette syndrome subjects were recruited from the outpatient clinic of the Department of Child and Adolescent Psychiatry at the Beijing Anding Hospital, Capital Medical University, China. Twenty TS subjects who met Diagnostic and Statistical Manual of Mental Disorders IV (DSM-IV) criteria (American Psychiatric Association, 2000) and 17 demographically matched healthy control children were enrolled in this study. The inclusion criteria for the TS group included right-handedness, treatment naïve and no other psychiatric diagnosis. Exclusion criteria for the control group included a lifetime history of tic disorder, OCD, ADHD, or a current DSM-IV Axis 1 disorder. Additional exclusion criteria for both groups were epilepsy, head trauma with loss of consciousness, former or present substance abuse, or an IQ below 70, as measured with the full scale Wechsler Intelligence Scale for Chinese Children-Revised (WISCC-R; Gong and Cai, 1993). All patients had normal neurological examinations except for their tics and were also evaluated with the Structured Clinical Interview for DSM-IV (SCID) to assess for possible comorbid psychiatric disorders. Tic severity was quantified using the Yale Global Tic Severity Scale (YGTSS; Leckman et al., 1989). All patients provided written informed consent for their participation in the study. Written informed consent was obtained from the next of kin, caretakers, or guardians on the behalf of the minors/children participants involved in this study. Regional Committee for Medical Research Ethics of Beijing Anding Hospital, Capital Medical University, approved the study.

### DATA ACQUISITION

Magnetic resonance imaging data were acquired on a 3.0 T SIEMENS TRIO scanner in the 306 hospital of the people’s liberation army of China. Participants were encouraged to minimize movements throughout the scan. Each Participant lay supine with the head snugly fixed by a belt and foam pads. In order to enhance participants’ abilities to maintain head position, a thermoplastic mask that molds to an individual’s face and attaches to the head coil was applied. Headphones dampened scanner noise and enabled communication with participants. High resolution anatomical images were obtained using 3D magnetization prepared rapid gradient-echo (MPRAGE) T1-weighted sequence with 176 sagittal slices, repetition time (TR) of 2530 ms, echo time (TE) of 3.39 ms, flip angle (FA) of 7°, voxel size of 1 mm × 1 mm × 1 mm. Resting-state functional images were acquired using single-shot gradient-echo echo-planar imaging (EPI) sequence with TR of 2000 ms, TE of 30 ms, FA of 90°, field of view (FOV) of 232 mm × 232 mm, and in-plane resolution of 64 × 64. Thirty-three 3 mm thick AC–PC parallel slices with 0.6 mm interslice gap were prescribed to cover the whole brain. During the rs-fMRI scans, the subjects were asked to remain still as much as possible, keep their eyes closed without falling asleep and try not to think systematically.

### DATA PREPROCESSING

Functional magnetic resonance imaging data were preprocessed using Statistical Parametric Mapping (SPM5)^1^ and REST toolbox^2^ (Song et al., 2011). The first 10 volumes of the functional images were discarded to equilibrate the signal and to allow participants’ adaptation to the scanning noise. Then functional images were corrected for slice timing and realigned for head motion correction. A 12-parameter affine transformation was estimated from each individual functional space into Montreal Neurological Institute (MNI) space, and the functional images were transformed to MNI space and re-sampled to 3-mm isotropic voxels. After smoothing with a 6 mm full-width at half maximum (FWHM) Gaussian kernel, the linear and quadric trends of time courses were removed. Several nuisance variables, including six head motion parameters, as well as the averaged signal from white matter and ventricles, were removed from the preprocessed time courses by multiple linear regression analysis.

### HEAD MOTION

To moderate the effects of head motion on ALFF, we first excluded three TS subjects and one control subject with excessive head movement > 3 mm maximum displacement in any of the x, y, or z directions or >3° of rotation in any direction. Moreover, given that “micro” head movements from one time point to the next can introduce systematic artifactual inter-individual and group differences in rs-fMRI metrics (Power et al., 2012; Van Dijk et al., 2012; Satterthwaite et al., 2013; Yan et al., 2013), it is recommended to scrub (remove or regress) time points with sudden movement on individual data (Yan et al., 2013). However, since the scrubbing procedure alters the underlying temporal structure of the data, precluding its application to frequency-based ALFF analysis(Yan et al., 2013), we chose to exclude the subjects who were of too much sudden micro head motion. For each subject, we counted the motion-contaminated fMRI volumes with framewise displacement (FD) > 0.5 mm and their one back and two forward neighbors. Subjects with less than 90 volumes (3 min) that were uncontaminated were excluded [one healthy control (HC) subject was excluded]. There were no significant differences in any of the six head motion parameters (all *p*s > 0.05) or the mean FD measure (*p* = 0.74) between the remaining 17 TS subjects and 15 HC subjects.

### ALFF AND fALFF ANALYSIS

The REST toolbox was used to calculate the ALFF/fALFF. After the above preprocessing, the fMRI data were temporally band-pass filtered (0.01–0.1 Hz) to reduce low-frequency drift and high-frequency respiratory and cardiac noise. The time courses were first converted to the frequency domain using a fast Fourier transform (FFT), and the power spectrum was obtained. The power spectrum obtained by FFT was square-rooted and then averaged across 0.01–0.1 Hz at each voxel. This averaged square-root was taken as the ALFF. To compute fALFF, the time courses were transformed into the frequency domain without band-pass filtering, and the sum of the amplitude across 0.01–0.1 Hz was divided by that of the entire frequency range (0–0.25 Hz). To reduce the global effects of variability across the participants, the ALFF/fALFF of each voxel was divided by the global mean ALFF/fALFF value within the entire brain. The global mean ALFF/fALFF was calculated only within the brain, with the background and other tissues outside the brain removed. These normalized ALFF and fALFF values were used for further statistical analyses.

### STATISTICAL ANALYSIS

Differences in demographic and clinical data between TS patients and healthy controls were analyzed using two-sample *t*-test and χ^2^-test in SPSS 16.0 (SPSS Inc., USA).

To investigate the differences in ALFF and fALFF between the two groups, a two-sample *t*-test was performed on the individual normalized ALFF and fALFF maps. To control for the possible influences of head motion on group-related results, we included individual mean motion estimates (i.e., mean FD) as a nuisance covariate (Van Dijk et al., 2012; Satterthwaite et al., 2013; Yan et al., 2013). Significant threshold was set at a corrected *p* = 0.05 combined with a cluster threshold of 117 voxels based on Monte Carlo simulations. To investigate the relationship between ALFF and clinical behavior (i.e., tic severity score), regression analysis was carried out between ALFF and the YGTSS scores with mean FD as a covariate in a voxel-by-voxel manner. The statistical threshold was set at a corrected *p* < 0.05 with a cluster threshold of 117 voxels.

## RESULTS

### DEMOGRAPHIC AND CLINICAL CHARACTERISTICS

**Table 1** shows the demographic and clinical characteristics of the TS patients and the healthy controls. The two groups were well matched in terms of age, sex distribution, years of education, and intelligence. All of the TS patients were drug-naïve and without any other psychiatric diagnosis including ADHD and OCD.

**Table 1:** Demographic and clinical characteristics.

Characteristics	TS patients (*n* = 17)	Controls (*n* = 15)	*p*-Value
Gender (male: female)	13:4	12:3	0.75
Age (year)	10.9 ± 3.0	10.8 ± 2.9	0.937
Education (years)	3.0 ± 1.8	2.5 ± 1.8	0.378
Full-scale IQ	103.2 ± 16.3	107.9 ± 16.1	0.420
Onset age (year)	5.6 ± 2.1	–	–
Disease duration (months)	47.8 ± 24.3	–	–
Tic severity score	37.7 ± 5.3	–	–

*Data are expressed as mean ± SD. SD, standard deviation; IQ, intelligence quotient; tic severity was evaluated by summing the motor tic and vocal tic sections of the YGTSS.*

### ALTERED ALFF/fALFF IN THE TS GROUP

**FIGURE 1:**
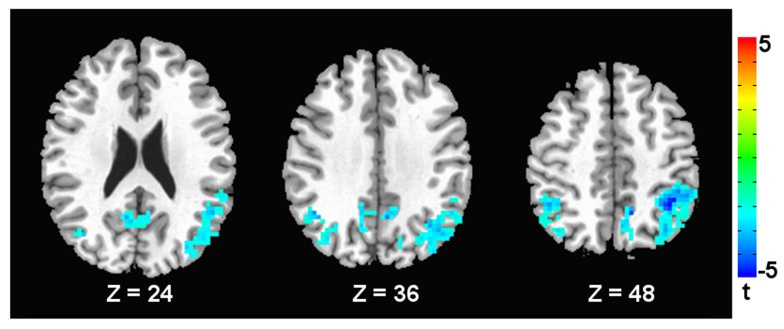
**Brain areas with decreased ALFF in TS children compared to healthy subjects.** A corrected significance level of *p* < 0.05 was obtained with a cluster size of 117 voxels. The left side of the image corresponds to the right side of the brain.

Compared with the healthy controls, the children with TS showed significantly decreased ALFF in the posterior cingulate cortex/precuneus (PCC/PCUN) and bilateral inferior and superior parietal lobules (**Figure 1** and **Table 2**).

**Table 2:** Brain regions showing significant between-group differences in ALFF.

Brain regions	Cluster size (voxels)	BA	MNI coordinates			*T* value
			*x*	*y*	*z*	
**TS < healthy controls**						
Posterior cingulate cortex/precuneus	1245	7	9	-54	42	-5.44
Right inferior parietal gyrus	201	40	45	-54	36	-3.85

*BA, Brodman area. *x*, *y*, *z*, coordinates of primary peak locations in the MNI space. The threshold was set at *p* < 0.05 (corrected, cluster size > 117 voxels).*

**FIGURE 2:**
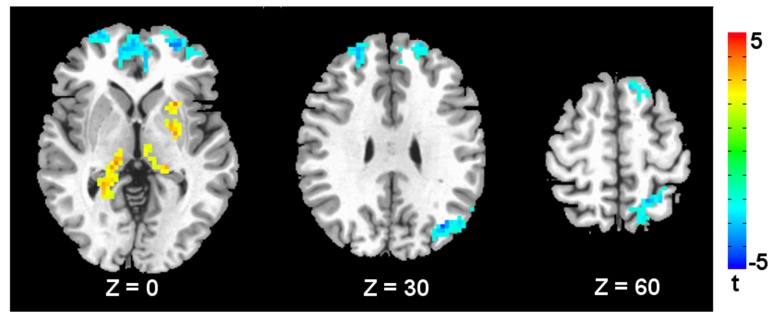
**Brain areas with decreased/increased fALFF in TS children compared to healthy subjects.** A corrected significance level of *p* < 0.05 was obtained with a cluster size of 117 voxels. The left side of the image corresponds to the right side of the brain.

**Figure 2** shows the fALFF differences between the TS patients and the healthy controls. Compared with the healthy controls, the children with TS showed significant fALFF decreases in the bilateral middle and superior frontal cortices, anterior cingulated cortex, left angular gyrus, and superior parietal lobule. The regions showing increased fALFF in the children with TS included the left putamen and bilateral thalamus. See **Table 3** for a list of these regions.

**Table 3:** Brain regions showing significant between-group differences in fALFF.

Brain regions	Cluster size (voxels)	BA	MNI coordinates			*T* value
			*x*	*y*	*z*	
**TS < healthy controls**						
Right middle and superior frontal gyrus	892	27	10	60	21	-5.45
Left angular gyrus	181	19	-54	-69	18	-5.02
Left superior parietal lobule	122	7	-21	-45	72	-4.27
Left superior frontal gyrus	120	9	-6	51	39	-3.66
**TS > healthy controls**						
Thalamus	229	–	15	-27	-3	4.44
Left putamen	134	–	-24	15	3	4.28

*BA, Brodman area. *x*, *y*, *z*, coordinates of primary peak locations in the MNI space. The threshold was set at *p* < 0.05 (corrected, cluster size > 117 voxels).*

### CORRELATIONS BETWEEN ALFF/fALFF AND TIC SEVERITY

**FIGURE 3:**
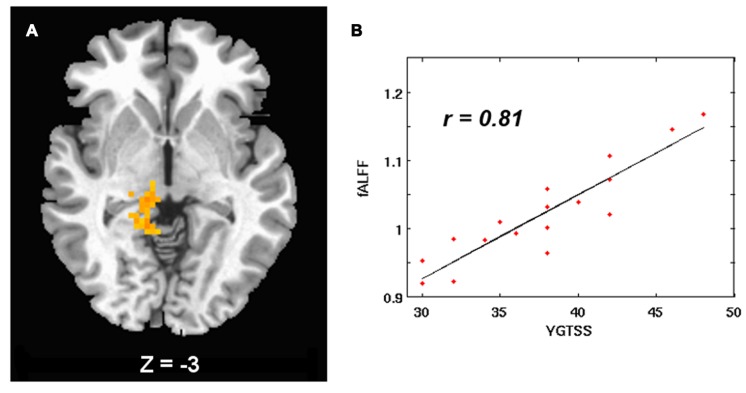
**(A)** fALFF positively correlated with tic severity in the right thalamus in TS children. Significant threshold was set at *p* < 0.05 with a cluster size of 117 voxels. The left side of the image corresponds to the right side of the brain. **(B)** Scatter plots show the relationship between tic severity and average fALFF in the regions of right thalamus.

In the children with TS, tic severity was positively correlated with fALFF in the right thalamus (**Table 4** and **Figure 3**).

**Table 4:** The relationship between ALFF values and tic severity in TS patients.

Brain regions	Cluster size (voxels)	BA	MNI coordinates			*T* value
			*x*	*y*	*z*	
Right thalamus	121	–	9	-48	0	7.42

*BA, Brodman area. *x*, *y*, *z*, coordinates of primary peak locations in the MNI space. The threshold was set at *p* < 0.05 (corrected, cluster size > 117 voxels).*

## DISCUSSION

Using ALFF/fALFF analysis on rs-fMRI data, we found abnormal spontaneous functional activity in TS patients. Compared with healthy controls, the children with TS exhibited significantly decreased ALFF in the posterior cingulate gyrus/precuneus and bilateral parietal gyrus. fALFF was decreased in TS children in the anterior cingulated cortex, bilateral middle and superior frontal cortices and superior parietal lobule, and increased in the left putamen and bilateral thalamus. We also found that fALFF in the left thalamus was positively correlated with tic severity in the TS children. The implication of these abnormalities for TS patients is discussed below.

We found significantly increased fALFF in the basal ganglia regions including the putamen and thalamus in the TS children group, which are important portions within the CSTC circuit. The model of dysfunction of CSTC in TS hypothesized that increased activity in striatal regions (i.e., caudate and putamen) inhibits the output nuclei of the basal ganglia and releases the thalamus from inhibition, which in turn excite the cortex to the production of tics (Mink, 2001). Previous fMRI studies have provided evidence in support of this model. For example, reduced activity in striatal regions and thalamus has been reported during tic suppression in TS children (Peterson et al., 1998), while increased activity of these regions has been found during the spontaneous tic performance in TS adults (Lerner et al., 2007; Wang et al., 2011a). Therefore, consistent with the dysfunction of the CSTC model, our results of increased fALFF in the striatal region of putamen may indicate that the excess activity in putamen disinhibits the thalamus, which also exhibited increased fALFF herein, and ultimately excites the cortex to yield unwanted tics. Furthermore, we observed that the increased fALFF in the thalamus was positively correlated with tic severity, confirming its putative role in the pathogenesis of TS.

We also found decreased fALFF in the middle and superior frontal gyrus and anterior cingulated cortex, and decreased ALFF and fALFF in the parietal gyrus in children with TS. These regions have been suggested to be involved in executive control functions (Petrides, 2005), and are associated with cognitive impairments in TS, including response inhibition, selective attention, and cognitive flexibility (Bornstein, 1990; Channon et al., 2003; Watkins et al., 2005). Previous fMRI studies in TS have demonstrated functional abnormalities in frontal and/or parietal areas. In children with TS, the prefrontal regions exhibited greater activation than in children without TS during a switching task (Baym et al., 2008; Jackson et al., 2011). Studies in TS adults have found that putative executive control regions, including the anterior cingulate, bilateral insula, and frontal and parietal regions, increased their activity prior to tic onset (Bohlhalter et al., 2006) and during tic suppression (Peterson et al., 1998), while exhibited less activity during spontaneous tic behavior (Wang et al., 2011a). In a recent rs-fMRI study, Church et al. (2009) investigated functional connections between regions within two control networks, i.e., the frontal-parietal network involved in adaptive online control and the cingulo-opercular network involved in stable set maintenance, and compared these connections in TS adolescents to typical developing subjects. They found significant immaturity in functional connections within both control networks, with frontal-parietal network experiencing the most abnormalities. Together with this evidence, our results of reduced ALFF/fALFF may be reflective of a disrupted cortical control circuit, which likely causes failure in inhibition of tic behaviors and abnormalities in cognitive control and executive functions in TS, and provide further support for the notion that TS model has been extended beyond CSTC circuitry (Martino and Leckman, 2013).

The children with TS have less intrinsic functional activity in the PCC/PCUN, which is a key region of the so-called default mode network (Raichle et al., 2001; Greicius et al., 2003). The posterior cingulate is thought to be involved in internally directed thought, such as retrieval of episodic and semantic memories (Gusnard et al., 2001; Buckner et al., 2008). More recently, PCC has also been demonstrated to play a role in regulating the balance between internal and external cognitive functions (Leech et al., 2012). Previous results show that the Tourette group failed to deactivate the PCC to the extent that healthy comparison subjects deactivated it during an eye blinking suppression task (Mazzone et al., 2010). In another study, default mode regions including PCC were found to show attenuated deactivations with age and associated with poorer performance in Tourette adults during a Stroop task (Marsh et al., 2007). The inability to deactivate the PCC in these studies suggests dysregulation between internal and external processing which may result in impaired abilities to suppress blink or to perform an executive task. In line with this evidence, our result of reduced intrinsic functional activity in the PCC/PCUN may represent functional disturbance in TS patients, which may lead to impaired regulation of tic and executive behaviors.

Abnormalities in intrinsic functional activity have previously been reported in TS comorbid disorders, including ADHD and OCD. For example, ADHD patients demonstrated aberrant ALFF in the anterior cingulate cortex, frontal and sensorimotor cortex (Zang et al., 2007; Yang et al., 2011), while OCD patients showed abnormal ALFF mainly in the anterior cingulate cortex and parietal cortex (Hou et al., 2012). However, studies of intrinsic functional activity during resting-state in these disorders are still limited, and it remains unclear whether TS subjects share the disruptions in the same or similar brain circuits.

### LIMITATIONS

The current study has several limitations. Firstly, given the relatively small sample size, further studies with a larger sample will be necessary to replicate, extend, and assess the generalizability of these findings. Secondly, we studied a highly selected patient population without clinically relevant psychiatric comorbidity and treatment history, in consideration that the pure TS sample could assure the resulting differences with healthy control are attributed to TS itself and not confounded by medication or comorbid disorders (Müller-Vahl et al., 2009; Thomalla et al., 2009). However, as we mentioned before, TS is strongly comorbid with ADHD (50% with TS also have ADHD) and OCD (20–60% with TS also have OCD; Singer, 2005), thus future studies using similar techniques should be carried out in TS patients with comorbidities and also OCD and ADHD patients without TS for comparison. Thirdly, our findings of the alterations in resting-state functional activity still need to be supported by future studies incorporating task design that allow for the identification of specific cognitive processes to further clarify the underlying components of these abnormal brain regions in TS individuals. For example, task design regarding executive control function such as stop-signal and task switching paradigm could help to understand the differences in frontal-parietal regions between TS and control group.

## CONCLUSION

In conclusion, the present study adopted the ALFF/fALFF approach on rs-fMRI data to investigate the alterations in spontaneous neural activity in TS patients. Abnormalities in TS children, including decreased ALFF in the posterior cingulate gyrus, bilateral parietal cortices, decreased fALFF in the anterior cingulated cortex, bilateral middle and superior frontal cortices and superior parietal lobule, and increased fALFF in the left putamen and bilateral thalamus, were consistent with previous model of dysfunction of CSTC circuit, and extend the disrupted anatomical pathology of TS to cortical control network and default mode network. The relationship between abnormal fALFF and the tic severity provided further support for our findings. These results shed light on the underlying neurophysiological mechanism reflected in the intrinsic brain activity in TS and demonstrate the feasibility of using the ALFF/fALFF as a research and clinical tool to access the dysfunction in TS.

## AUTHOR CONTRIBUTIONS

Yonghua Cui, Xu Chen, Yong He, Xia Liang, and Yi Zheng designed the study. Zhen Jin and Xu Chen acquired the data. Yonghua Cui, Xu Chen, and Xia Liang analyzed the data. Yonghua Cui and Xia Liang wrote the article. Yonghua Cui, Zhen Jin, Xu Chen, Yong He, Xia Liang, and Yi Zheng reviewed the article. All authors approved its publication.

## Conflict of Interest Statement

The authors declare that the research was conducted in the absence of any commercial or financial relationships that could be construed as a potential conflict of interest.

## Footnotes

1 http://www.fil.ion.ucl.ac.uk/spm/

2 http://resting-fmri.sourceforge.net
